# Obstetric Considerations in a Rare Cardiovascular Catastrophe Needing Multidisciplinary Care

**DOI:** 10.1155/2014/278036

**Published:** 2014-03-04

**Authors:** Neeta Singh, Debjyoti Karmakar, V. Devagorou, Rajnish Tiwari, Sunesh Kumar

**Affiliations:** ^1^Department of Obstetrics and Gynaecology, All India Institute of Medical Sciences, Ansari Nagar, New Delhi 110029, India; ^2^Department of Cardio Thoracic and Vascular Surgery, Cardio Thoracic Centre, All India Institute of Medical Sciences, New Delhi 110029, India

## Abstract

Cardiovascular emergencies especially aortic dissections are rare in pregnancy. We report a case of Stanford Type A aortic dissection at 33 weeks of pregnancy presenting in shock. Rapid multidisciplinary approach and special obstetric considerations led to a successful outcome in this case.

## 1. Introduction

Cardiovascular emergencies needing precipitous decision making is somewhat rare in pregnancy. Only about 5% of nontraumatic sudden deaths in the young population are caused by aortic dissections and it is even less common in females [1, 2]. The majority are associated with connective tissue disorders [3]. As such, obstetricians rarely encounter the much-dreaded dissections of ascending aorta (Stanford Type A) [3]. We present one such case managed successfully at our institution.

## 2. Case Summary

**Figure 1:**
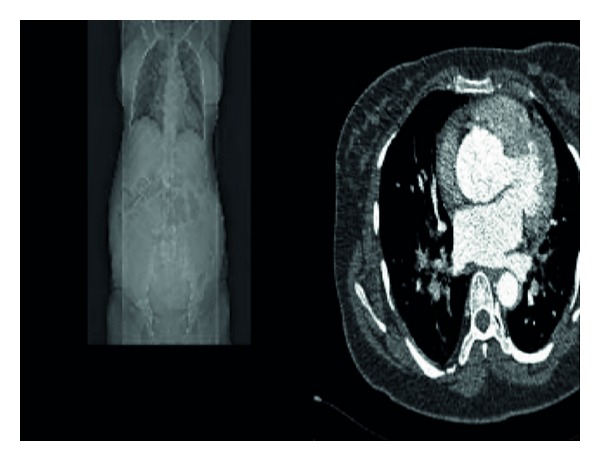
Computerised tomography angiography of the patient revealing a massive type A aortic dissection of the thoracic aorta extending proximally from the aortic root to proximal arch with extension into proximal innominate artery.

A 28-year-old, previously healthy, third gravida at 33 weeks and 4 days with previous two vaginal deliveries presenting with syncope, chest pain, profound hypotension, and weakness of right arm at the Emergency Department. CT angiogram, following an Emergency Medicine consult, revealed a massive type A aortic dissection of the thoracic aorta extending proximally from the aortic root to proximal arch with extension into proximal innominate artery (Figure 1). Her syphilis serology was normal. There were no stigmata of Marfan's syndrome and emergent Trans Thoracic Echo did not reveal any underlying congenital cardiac malformation. Her height was 156 cm and weight 63 kg. Repair of aorta was planned after initial stabilization and obstetrics consult sent for cesarean delivery immediately preceding it. She received two doses of antenatal corticosteroids (betamethasone) for fetal lung maturity, second dose being on the day of surgery. Perioperatively, the patient was heparinised and monitored by Trans Esophageal Echocardiogram (TEE) and general anesthesia given in left lateral tilt. Lower Segment Cesarean Section (LSCS) with tubal sterilization was done with controlled and gradual fetus extraction to avoid sudden abdominal decompression. While the heparinisation added to the risk of intraoperative and obstetric hemorrhage, avoiding bleeding in postpartum period was of utmost priority in this patient. Oxytocin could precipitate hypotension and prostaglandins are unsafe because of the cardiovascular considerations. Also it was prudent to avoid vasopressors in case she did have obstetric hemorrhage. Prophylactic bilateral uterine artery ligation was done and 20 U Oxytocin was added to 500 mL Ringer's lactate and then tapering over an extended period of 48 hours. Thereafter aortic repair was done by Bentall procedure (Dacron graft) with fixation of dissection at base of innominate artery under cardiopulmonary bypass. The repair was accomplished in 46 hours from time of presentation to the emergency. She received 7 units of packed red blood cells and stood the procedure well. She was kept in ICU for 48 hrs and then shifted to ward on the third day. The baby at birth was 2.4 kg and did well in neonatal period.

## 3. Discussion

In an aortic dissection, apart from the calamitous cardiovascular compromise of the mother, the additional anesthetic interventions such as left ventricular assists and cardiopulmonary bypass also add risk of fetal death if repair is done before delivery of fetus [3, 4]. This communication tries to highlight the special obstetric considerations to this rare situation. The treatment of type B dissections (involving the aorta distal to the origin of the left subclavian artery) is mainly medical [3]. Type A dissections have a maternal mortality rate of 1% per hour and always require emergency surgery with the fetus kept in utero during repair before 28 weeks [5]. After 32 weeks gestation primary cesarean section is followed by aortic repair in the same sitting [5]. The rest of the cases need individualization. The risk of death is as high as 30% for fetus [5].

This aortic vascular catastrophe led to a decision of preterm LSCS with uterine artery ligation in a multiparous lady with previous vaginal deliveries after administration of antenatal corticosteroids with additional efforts to prevent obstetric hemorrhage during and after surgery. At the time of cesarean delivery care must be taken to extract fetus gradually out of abdomen while avoiding fundal pressure [3, 4]. The presence of a skilled and experienced obstetricians and neonatologists is absolutely prudent to ensure fetomaternal wellbeing in the face of this very rare and life threatening emergency in this subset of patients. It was paramount to expedite the repair in the best interests of the mother considering the time dependent mortality mentioned above. Active multidisciplinary involvement with specialty consultants taking the lead led to the diagnosis, stabilization, and complication free multimodality management within a competitive timeframe, ensuring a successful outcome. There are very few reports of successful Bentall repair of aorta with composite graft replacement of the aorta and aortic valve in peripartum period [3–5].

We want to highlight that in a calamitous situation like this, a preoperative multidisciplinary discussion between cardiothoracic surgeons, cardiac anesthetics, obstetricians, and neonatologists is of paramount significance and this was an integral part of successful management of our patient as well. We had to incorporate unique obstetric considerations in this special situation to salvage the mother.
